# Notes on the Purification and Properties of Chloroform*Although I am alone responsible for the opinions contained in this paper, it is my duty to state, that all the experiments and observations mentioned in it have been made by me in concert with my able assistant, Mr. Alexander Kemp, of whose ingenuity and accuracy I have had constant opportunities of judging.

**Published:** 1850-12

**Authors:** 


*Notes on the Purification and Properties of Chloroform.** By WILLIAM GREGORY, M. D., Professor of Chemistry in the University of Edinburgh.†

1. Chloroform has been prepared both from alcohol and wood-spirit. The latter has been used for the sake of cheapness; but as it is a mixture of several liquids, all of which do not yield chloroform, it gives an impure product, in a proportion which varies much, but is always below that obtained from alcohol. There is, therefore, not only no advantage, but the contrary, in using wood-spirit, which is not, after all, much cheaper than alcohol.

2. But the chloroform from these two liquids, *when fully purified*, is quite identical in all its properties. Its smell, density, boiling-point, and action in the system, are in both cases exactly the same. That from alcohol is no doubt more easily purified than the other, but it also contains certain volatile oily impurities, which must be removed before it can be safely used. The peculiar oils which adhere to both kinds of chloroform are not identical, or at least, not all identical, but they are of analogous constitution and properties.

3. Soubeiran and Mialhe have examined these oils. They contain chlorine, have a disagreeable smell, and when inspired or smelt, cause distressing headache and sickness. In the case of wood-spirit, some of its own impurities distil over unchanged, and are also found in the chloroform.

4. It is well known that many persons, after the use of chloroform, have suffered from headache, nausea, and even vomiting, as I have more than once seen. Headache and nausea I have myself often experienced, when I have tried different specimens of chloroform, without taking so much as to produce the full effect.

5. Perfectly pure chloroform does not, so far as I have seen or experienced, produce these disagreeable effects. It is therefore highly probable, that when they occur, as they do with some individuals, from the use of chloroform of more than the average goodness of quality, they depend on the presence of a trace of these poisonous oils.

6. All good manufacturers of chloroform, purify it by the action of oil of vitriol, which destroys the oils, while at the same time a part of the acd is reduced to sulphurous acid. The chloroform, to remove this, is then distilled with lime or carbonate of baryta, and is tolerably pure if the process be well conducted.

7. But it is not quite pure, and contains a trace, more or less distinct, of the oils. I have found this to be the case with all the best chloroform made here up to 1849; and I have several times seen headache and sickness from the use of such chloroform, which was the best anywhere made. I must add, however, that the quantity of oils in the chloroform of the best Edinburgh manufacturers, although variable within certain limits, was always so small, that that product was fit for use, and only caused headache, &c., in a few peculiarly sensitive persons.

8. It was desirable to have a test for these impurities, as well as an easy and effectual mode of removing the last traces of them, especially as many

* Although I am alone responsible for the opinions contained in this paper, it is my duty to state, that all the experiments and observations mentioned in it have been made by me in concert with my able assistant, Mr. Alexander Kemp, of whose ingenuity and accuracy I have had constant opportunities of judging.

† Read before the Royal Society of Edinburgh, March, 1850.

sorts of chloroform not made here were far inferior in quality to that prepared in Edinburgh. One very delicate test is, that of oil of vitriol, which should be quite colorless, pure, and of the full density of 1.840 at least, as it may be obtained by Mr. Kemp's process, lately read to the Royal Society; when agitated with the chloroform, it becomes yellow or brown, from its action on the oils, which it chars or destroys. Any change of color is easily seen by contrast with the colorless chloroform which floats above. Pure chloroform gives no color to the acid. It is essential that the oil of vitriol be colorless and also of full density; for if colored, it is not easy to see a slight change on its color; and if below the proper density, that is too weak, it is not much colored by a chloroform which will render dark brown the acid of proper strength.

9. Another test, still more delicate, I find to be the smell of the oils. When chloroform is poured on the hand or on a handkerchief, it rapidly evaporates; but the oil being less volatile, are left behind; and their smell, previously covered by that of the chloroform, is easily recognized. Until very lately no chloroform was sold, or indeed known, which would stand this test, or even the former.

10. Up to 1849 the best commercial chloroform had a specific gravity of 1.480, which was considered a guarantee of its purity; but it had been obtained by chemists of specific gravity 1.494, and even 1.497. I have found that chloroform of 1.480, when once more acted on by oil of vitriol, which destroys the oils and becomes brown, may be obtained after removing the sulphurous acid, of specific gravity 1.500 at 60°. This I take to be the specific gravity of pure chloroform. Our best makers have lately, much to their credit, pushed the purification so far as to furnish chloroform even of this highest density, and also in other respects such as it ought to be.

11. There are still, however, many makers in other places whose chloroform is not so pure; and I shall now describe the method which, with Mr. Kemp, I have employed for purifying, perfectly and easily, any commercial chloroform, except one remarkable specimen—a process which will enable any medical man to purify it for himself with the greatest facility.

12. The chloroform, having been tested as above, and found more or less impure, is to be agitated with the oil of vitriol, (half its volume will be sufficient,) and *allowed to remain in contact* with the acid, of course in a clean, dry, and stoppered bottle, and with *occasional agitation* till the acid no longer becomes darker in color. As long as the action is incomplete, there will be seen, after rest, at the line of contact, a darker ring. When this no longer appears, the chloroform may be drawn off, and for greater security once more acted upon by a quarter of its volume of the acid, which should now remain colorless. It is now to be once more drawn off, and in a dry stoppered bottle mixed with a little powdered peroxide of manganese, with which it is gently agitated, and left in contact until the odour of sulphurous acid is entirely destroyed, and the chloroform has acquired a mild, agreeable, fruity smell. It has then only to be poured off into a proper phial. It will now leave no disagreeable smell when evaporated on the hand. [If the commercial chloroform, after having been *frequently well shaken* and *left for some time in contact* with the acid, has given to it only a moderate tinge of color, it is probable that it may be completely purified by the first process. To ascertain this, test a fresh portion in a tube with fresh acid, shaking well and allowing it to stand for some time. If it do not color the acid at all, then the whole chloroform has only to be finally purified by the oxide of manganese. If the acid become colored in the test-tube, it will be as well to act on the whole chloroform a second time with fresh acid till it stands the test. Mr. Kemp has observed, in repeating this process for me, the very curious fact that, as soon as the action is complete, and the oily impurities are destroyed, but not sooner, the chloroform tested with the acid in a tube, exhibits a strongly convex surface downwards, where it rests on the pure acid, or what is the same thing, the acid becomes concave at its upper surface. The smallest trace of impurity, not sufficient to affect the density of the chloroform, we have found to render the line of junction horizontal. It is probable that this may become a valuable test of the perfect purity of chloroform; but we shall not say more on this subject until we have thoroughly examined it.] This process requires no apparatus beyond a few stoppered bottles and a *pipette,* if we wish to draw off the whole chloroform without loss, although nearly the whole may be simply poured off. The use of the oxide of manganese is due to Mr. Kemp; and on the large scale the chloroform may be filtered through a cylinder full of it. In this final purification of commercial chloroform, no distillation is necessary. Indeed, no rectification is required at all, if it be well washed with water before using the acid.

13. It may be considered as certain, that the use of chloroform thus purified, will very rarely, if ever, cause the disagreeable effects above noticed.* As to more serious bad results from the use of chloroform, so often spoken of elsewhere, it is enough to state that a large proportion of the cases must be attributed to the use of a liquid so impure as hardly to deserve the name of chloroform at all.

*Postscript.*—Since writing the above, my attention has been called to a paper by Dr. Wilson, on the specific gravity of chloroform, which he was not able to obtain higher than 1.498. I have therefore to add, that every specimen, whether of specific gravity 1.480, 1.490, or 1.497, which I purified as above, acquired the same density of 1.500, as ascertained by the use of a very delicate and accurative bead, (made by Lovi,) which sank at 60.°5

* Dr. Simpson informs me, that the purest chloroform he has used not unfrequently causes vomiting. On further inquiries, I find that this occurs when it is administered after a full meal. This can easily be avoided, and must not be confounded with the headache, nausea and vomiting alluded to in sections 4 and 5. which symptoms are persistent, and occurred in my experiments always with an empty stomach, the experiments being made an hour or two before dinner. Mr. Carmichael, assistant to Dr. Simpson, has mentioned to me some facts which confirm the view I have taken. At one period, for more than a week, Dr. Simpson and Mr. Carmichael were kept in a state of continual anxiety by the occurrence, in all the puerperal cases in which chloroform was used, of very unpleasafit symptoms, particularly of frequent pulse and other febrile symptoms, lasting for some days. At last, after much annoyance from this cause, it occurred to Dr. Simpson that he was using one particular specimen of chloroform above the average in quality. As soon as this idea occurred, he threw away all that remained, and returned to that which he had generally used. The unpleasant symptoms no longer appeared. (I regret much that I had not an opportunity of examining that specimen; but I may add, that the maker, not an Edinburgh one, now produces chloroform of much better quality, though not yet absolutely pure.) But the striking fact is this, that Dr. Simpson and Mr. Carmichael state, that during the period above alluded to, when that one kind of chloroform alone was used by them, their handkerchiefs became quite offensive from the smell left on them, which even adhered to them after washing. There can, I think, be no doubt, that here the oily impurities alluded to in sections 4 and 5 were present in notable quantity.

and rose at 59.°5; and also by three successive weighings with a very delicate balance. It will also be seen, that three commercial specimens had this density; I could detect no foreign matter in my chloroform; and besides, every foreign matter that is likely to occur *lowers* the density. I have no doubt that Dr. Wilson's specimens would have colored the acid and left a smell on the hand.

I may add, for the maker, that, after distilling the materials which yield chloroform, no distillation or rectification is needed. He has only to wash the heavy fluid with water till its volume no longer diminishes, and then to use the oil of vitriol as above, finishing with the oxide of manganese. Distillation with the acid is of no use, because no proper contact can take place, the chloroform distilling from the surface as it would from mercury. In testing by oil of vitriol, it is best to use some ounces of chloroform, and to shake it in a phial, because in a test-tube, the color produced, if not strong, may be overlooked.

While I acquit the makers of chloroform, who have sold an impure drug, of all desire or intention to adulterate it, I feel it my duty to point out, that the system which permits *any one* to set up as a manufacturer of this or any other potent remedy, without let or hindrance, without any test of his qualifications, without, in short, enforcing a knowledge of chemistry and pharmacy as an essential condition, is a radically bad one; and that our law, in relation to Druggists and Apothecaries, requires reformation. In fact, the evils naturally resulting from it are only neutralized, and that but in part, by the good feeling and principle of the leading manufacturers.

To illustrate this, I may remark, that some of the makers of chloroform must have been very ignorant, even of what was known and published concerning its properties; for, among the specimens I examined, and several of specific gravity below 1.480, which was long ago given as the standard, even so low as 1.347.

That this neglect proceeded more from ignorance than from intention, is, I think, plain from the fact, that a specimen labelled "Pure Chloroform" actually contained only a trace, about one-thirtieth, of that substance. I did not ascertain its specific gravity, which must have been far lower than 1.200 or 1.100—nay, possibly, under 1.000, because its impurity was so obvious in every other respect, and the quantity I had was too small; but, on examining it further, I am convinced that its origin was this:—The maker, after distilling the materials, obtained, of course, two liquids, a lighter and a heavier. He evidently *did not know* that the latter was the chloroform, and therefore threw it away, and preserved the *lighter*—a mixture of pyroxilic spirit—of its natural impurities, of the deleterious chlorinated oils and a *trace* of chloroform. At least, such are its characters; and it exactly resembles what would be obtained in the way supposed. But what a fearful degree of ignorance (without any evil intention) is here exhibited! And yet this maker was free to produce and sell *pure chloroform*, which was actually almost *pure from chloroform*, and loaded with deleterious agents.—*Monthly Journal of Medical Science*, May, 1850, *and Pharm. Jour.* June, 1850.

[A few weeks since, having occasion for some chloroform, we obtained a pound from a manufacturing establishment in this city. On opening the bottle the odor of the chloroform was contaminated with that of chlorine, and the stopper had on it a greenish yellow discoloration. On returning it to the chemist with the information of its impurity, we received the following note:

"Dear Sir: We have your favor of this morning, in regard to chlorine found in chloroform, and for the same are much obliged, as it gives us some ground, other than our own opinion, for declining to make the article by the late improved process with sulphuric acid. We soon discovered that this change was likely to go on, but some of the customers wanting an article of this kind (*i. e.* made by this process) particularly, we were induced to adopt the improvement."

This chloroform bleached moist litmus paper rapidly, first reddening it. The acidity is due to hydrochloric acid, generated from the chlorine and moisture adhering to the chloroform operated on by light. Mr. Abraham, in the paper, at page 348, has arrived at similar conclusions.—EDITOR.]—*Med. Jour. of Phar.*

